# Mimetics of extra virgin olive oil phenols with anti-cancer stem cell activity

**DOI:** 10.18632/aging.202154

**Published:** 2020-11-09

**Authors:** Elisabet Cuyàs, Juan Gumuzio, Jesús Lozano-Sánchez, Antonio Segura-Carretero, Sara Verdura, Joaquim Bosch-Barrera, Begoña Martin-Castillo, Alfons Nonell-Canals, Amadeu Llebaria, Silvia Cabello, Carme Serra, Melchor Sanchez-Martinez, Ángel G. Martin, Javier A. Menendez

**Affiliations:** 1 Program Against Cancer Therapeutic Resistance (ProCURE), Metabolism and Cancer Group, Catalan Institute of Oncology, Girona, Spain; 2 Girona Biomedical Research Institute (IDIBGI), Girona, Spain; 3 StemTek Therapeutics, Bilbao, Spain; 4 Research and Development of Functional Food Centre (CIDAF), Granada, Spain; 5 Department of Food Science and Nutrition, University of Granada, Granada, Spain; 6 Department of Analytical Chemistry, University of Granada, Granada, Spain; 7 Medical Oncology, Catalan Institute of Oncology, Girona, Spain; 8 Department of Medical Sciences, Medical School University of Girona, Girona, Spain; 9 Unit of Clinical Research, Catalan Institute of Oncology, Girona, Spain; 10 Mind the Byte, Barcelona, Spain; 11 MCS, Laboratory of Medicinal Chemistry and Synthesis, Institute of Advanced Chemistry of Catalonia (IQAC-CSIC), Barcelona, Spain; 12 SIMChem, Synthesis of High Added Value Molecules, Institute of Advanced Chemistry of Catalonia (IQAC-CSIC), Barcelona, Spain; 13 Molomics, Barcelona Science Park, Barcelona, Spain; 14 Current address: The Patients Resource, Barcelona, Spain

**Keywords:** olive oil, mTOR, DNMT, metabolism, epigenetics

## Abstract

The extra virgin olive oil (EVOO) dihydroxy-phenol oleacein is a natural inhibitor of multiple metabolic and epigenetic enzymes capable of suppressing the functional traits of cancer stem cells (CSC). Here, we used a natural product-inspired drug discovery approach to identify new compounds that phenotypically mimic the anti-CSC activity of oleacein. We coupled 3D quantitative structure-activity relationship-based virtual profiling with phenotypic analysis using 3D tumorsphere formation as a gold standard for assessing the presence of CSC. Among the top 20 computationally-predicted oleacein mimetics, four fulfilled the phenotypic endpoint of specifically suppressing the tumorsphere-initiating capacity of CSC, in the absence of significant cytotoxicity against differentiated cancer cells growing in 2D cultures in the same low micromolar concentration range. Of these, 3,4-dihydrophenetyl butyrate –a lipophilic ester conjugate of the hydroxytyrosol moiety of oleacein– and *(E)-N*-allyl-2-((5-nitrofuran-2-yl)methylene)hydrazinecarbothioamide) –an inhibitor of *Trypanosoma cruzi* triosephosphate isomerase– were also highly effective at significantly reducing the proportion of aldehyde dehydrogenase (ALDH)-positive CSC-like proliferating cells. Preservation of the mTOR/DNMT binding mode of oleacein was dispensable for suppression of the ALDH^+^-CSC functional phenotype in hydroxytyrosol-unrelated mimetics. The anti-CSC chemistry of complex EVOO phenols such as oleacein can be phenocopied through the use of mimetics capturing its physico-chemical properties.

## INTRODUCTION

Extra virgin olive oil (EVOO) is a unique functional food with a major contribution to the health-promoting effects of the so-called Mediterranean diet. EVOO contains a group of complex phenol-conjugated compounds named oleosidic secoiridoids or oleosides that exert nutritional and beneficial effects on major aging-driven diseases including cancer [1–10]. Using a holistic approach for phenotypic drug discovery coupled with mechanism-of-action functional profiling and target deconvolution, we recently identified the dihydroxy-phenol oleacein (the dialdehydic form of decarboxymethyl elenolic acid linked to hydroxytyrosol) [11–17] as a metabolo-epigenetic inhibitor of the mammalian target of rapamycin (mTOR) kinase and DNA methyltransferases (DNMTs). Oleacein was found to specifically and potently suppressing the functional traits of tumor-initiating cancer stem cells (CSC) in genetically diverse types of cancer cell populations [18].

The anti-CSC effects of oleacein are most likely related to its chemical structure, largely due to the presence of two hydroxyl groups in the hydroxytyrosol moiety [9, 19–21]. Therefore, one could envision that its scaffold might be used as a chemical prototype to facilitate selection and advancement of new anti-CSC hits via cell-based phenotypic screenings. However, a recent delineation of the high-level functions of oleacein in terms of biomolecular interactions, signaling pathways, and protein-protein interaction networks revealed that the so-called oleacein target landscape likely involved more than 700 proteins rather than solely mTOR and DNMTs [22]. Thus, although the ability of oleacein to operate as a multi-faceted regulator of numerous metabolic processes and chromatin-modifying enzymatic activities might open new horizons for CSC-targeted therapy based on the molecular bridge that connects metabolism and epigenetics with the aberrant state of stemness in cancer tissues [23–28], a biomimicry design process of oleacein mimetics remains a highly challenging task.

**Figure 1:**
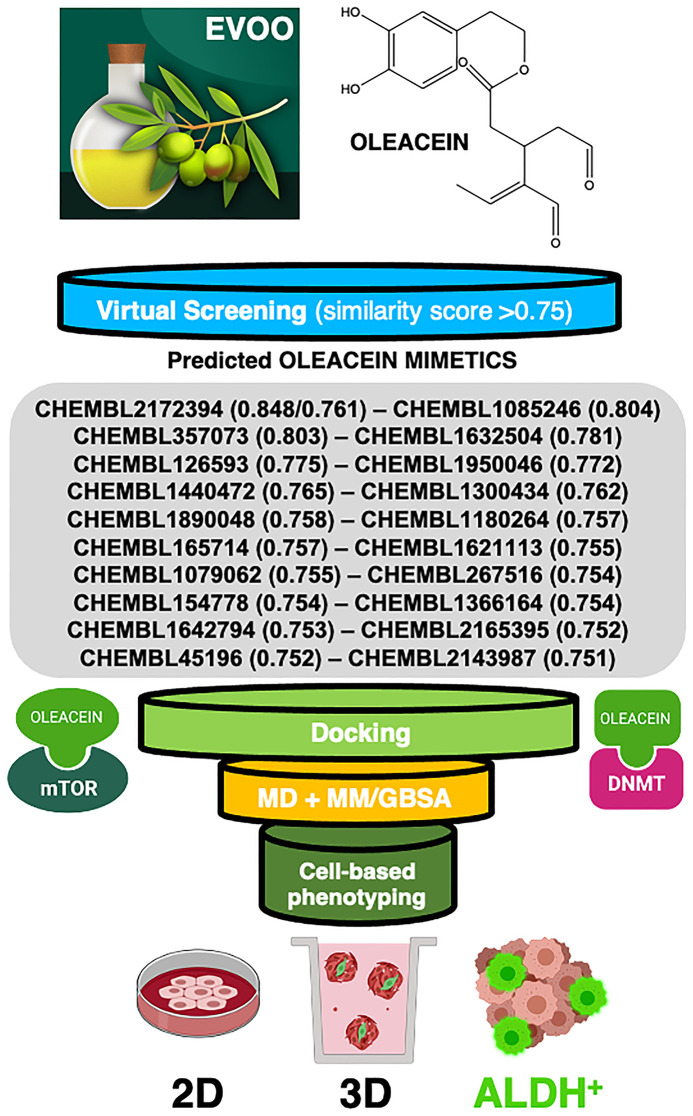
**Computer-assisted discovery of oleacein biomimetics with anti-CSC activity.** Schematic illustration of the computational framework coupled to laboratory-based phenotypic testing. The values in parentheses are similarity scores calculated with respect to parental oleacein.

Here, we used a natural-product-inspired drug discovery approach to identify new small molecules capable of phenotypically mimicking the anti-CSC actions of oleacein. Using the structure of oleacein as a “seed”, we coupled 3D quantitative structure-activity relationship (3D-QSAR)-based virtual profiling (VP) with laboratory-based phenotypic testing using tumorsphere-formation potential as a gold standard for evaluating the presence of CSC (Figure 1). We provide evidence that oleacein can be phenocopied through the use of mimetics with anti-CSC activity, which might guide the design of synthetically tractable small molecules capable of phenotypically imitating the anti-CSC chemistry of complex EVOO phenolics.

## RESULTS

### Computer-assisted discovery of oleacein mimetics

When a 2D similarity, ligand-based VP program was executed over the Chembl(v19) database using the Tanimoto coefficient and 2D (Morgan/circular) fingerprints, only the closely related secoiridoid molecule oleocanthal (CHEMBL2172394) was identified. The execution of a comparative molecular similarity indices analysis (CoMSIA)-based 3D VP program, however, identified several compounds with physico-chemical similarity scores greater than 0.75 (Figure 1). Taking advantage of the previously described binding modes of oleacein to mTOR and DNMT [18], we ran rigid-docking calculations to characterize the binding modes of the top 20 oleacein mimetics (Supplementary Figure 1), both at the crystallographic sites and at additional cavities occurring within the whole protein structures of mTOR and DNMT (Supplementary Tables 1–3). Table 1 summarizes the computationally-predicted oleacein mimetics ranked according to reweighted energies based on short molecular dynamics (MD) simulations followed by molecular mechanics with generalized Born and surface area solvation (MM/GBSA) calculations, for both the crystallographic and the best mTOR/DNMT cavities for each of the selected oleacein mimetics (Supplementary Tables 4 and 5).

**Table 1:** MM/GBSA-based binding energy rescoring calculations over MD simulations of computationally-predicted oleacein mimetics.

**Candidates ranked by MM/GBSA energy 4JT6 (mTOR)**	**MM/GBSA energy Crystallographic cavity / Best cavity**	**Candidates ranked by MM/GBSA energy 4WXX (DNMT)**	**MM/GBSA energy Crystallographic cavity / Best cavity**
**oleacein**	-26.8226 / -36.9331	**oleacein**	-30.567 / -36.5163
**CHEMBL1300434**	-38.7014 / -27.361	**CHEMBL1632504**	-38.2609 / -36.6319
**CHEMBL2143987**	-32.4070 / -40.3344	**CHEMBL2143987**	-36.4821 / -43.6863
**CHEMBL1545778**	-30.5493 / -25.0387	**CHEMBL2165395**	-33.4134 / -25.8227
**CHEMBL126593**	-29.2106 / -26.6329	**CHEMBL1300434**	-33.3421 / -33.9773
**CHEMBL1085246**	-27.4436 / -19.6725	**CHEMBL267516**	-32.8788 / -28.1508
**CHEMBL267516**	-27.3710 / -44.6454	**CHEMBL1180264**	-31.7196 / -32.3981
**CHEMBL45196**	-27.2624 / -17.1961	**CHEMBL357073**	-28.4676 / -27.0541
**CHEMBL1632504**	-25.7896 / -24.6272	**CHEMBL1440472**	-27.5899 / -29.3600
**CHEMBL357073**	-25.0102 / -33.5462	**CHEMBL1621113**	-26.6488 / -29.3269
**CHEMBL1366164**	-24.3303 / -17.8085	**CHEMBL1890048**	-26.0912 / -26.2952
**CHEMBL1642794**	-24.1435 / -19.439	**CHEMBL126593**	-25.7134 / -35.3592
**CHEMBL1621113**	-22.9663 / -21.0309	**CHEMBL45196**	-24.5175 / -32.1555
**CHEMBL1950046**	-20.2999 / -31.6794	**CHEMBL1950046**	-24.3167 / -21.7283
**CHEMBL2165395**	-19.8235 / -27.2639	**CHEMBL1079062**	-24.2025 / -24.4205
**CHEMBL1890048**	-19.6392 / -21.2089	**CHEMBL1545778**	-21.6215 / -22.9832
**CHEMBL2172394**	-18.4177 / -34.4392	**CHEMBL1085246**	-17.8140 / -21.8923
**CHEMBL1180264**	-18.2272 / -29.4140	**CHEMBL1642794**	-16.1264 / -20.6555
**CHEMBL1079062**	-17.4413 / -24.7585	**CHEMBL1366164**	-15.4957 / -19.6201
**CHEMBL1440472**	-16.6468 / -21.2853	**CHEMBL165714**	-12.1247 / -30.3770
**CHEMBL165714**	-16.1321 / -21.4634	**CHEMBL2172394**	-11.8887 / -31.0757

### Binding modes of oleacein mimetics to mTOR and DNMT

**Figure 2:**
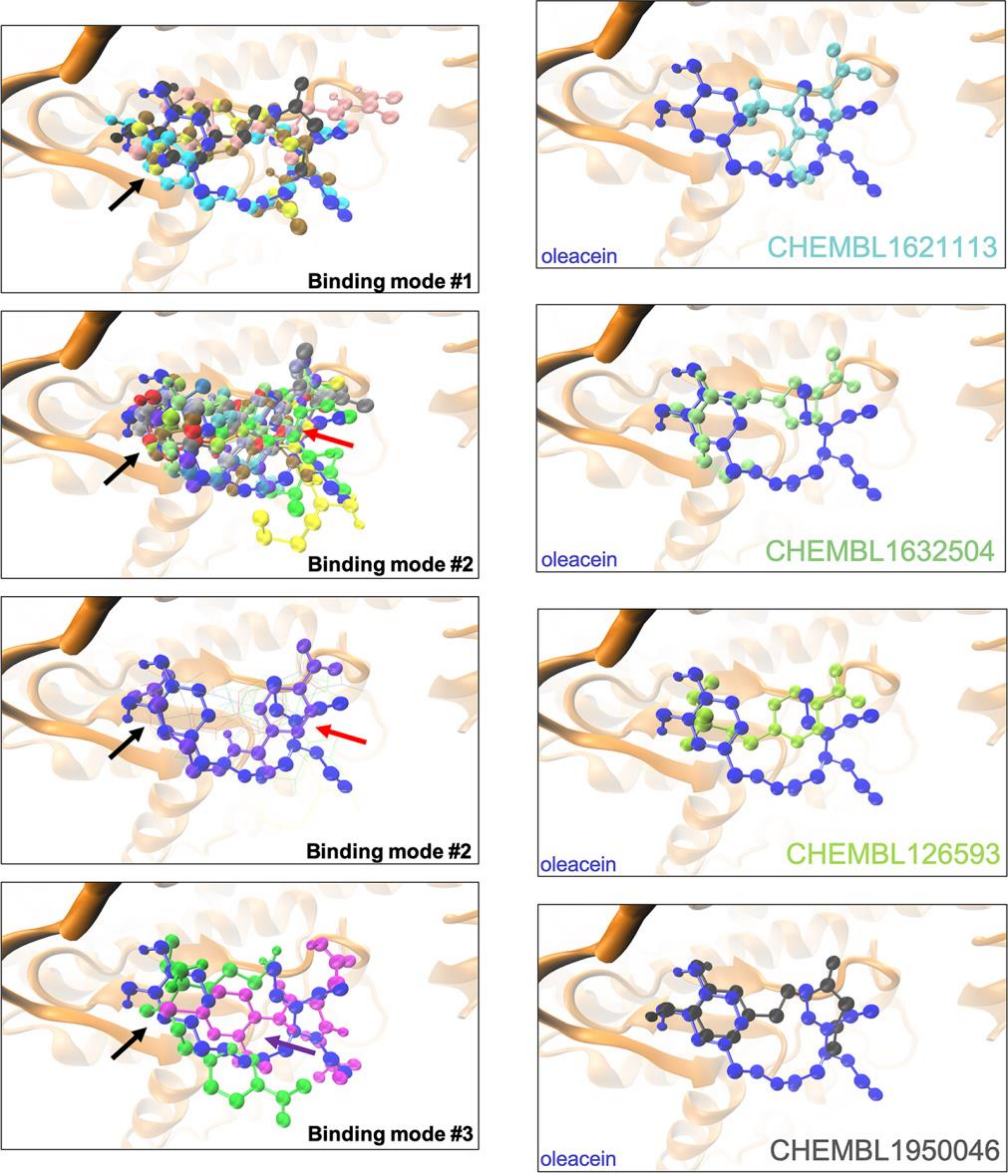
**Binding modes of oleacein mimetics to mTOR. *Left panels.*** Graphical representation of the binding modes of the computationally-predicted oleacein mimetics to the catalytic cavity of mTOR. The black, red, and purple arrows indicate the location of the aromatic rings in the binding modes #1, #2, and #3, respectively. *Right panels.* Graphical representation of the binding modes of parental oleacein and selected oleacein mimetics with anti-CSC activity (Figure 4, 5) to the catalytic cavity of mTOR.

The binding mode of oleacein to mTOR was predicted to share key amino acid residues with the binding modes of second-generation ATP-competitive TORKinhibs and, consequently, partially mimicked the binding behavior of PP242 and Torin 2 to the ATP-binding catalytic pocket [18]. But, the presence of more aromatic rings in the oleacein molecule resulted in a slightly different binding strength from that of PP242 and Torin 2. Similarly, the presence of aromatic rings notably influenced the binding of the selected oleacein mimetics to mTOR (Figure 2). In fact, we predicted three different binding modes, one of them involving 5 oleacein mimetics that apparently shared the originally described binding mode of parental oleacein; and another two models encompassing 13 compounds and 2 compounds showing a binding mode closely resembling that of TORKinhibs (Figure 2). Rigid docking calculations originally predicted that π-π stacking would occur between the aromatic ring of oleacein and the Trp2239 residue (or Tyr2225 upon conformational changes of either oleacein or the mTOR catalytic pocket itself) in the catalytic site of mTOR. MD simulations confirmed the main occurrence of π-π stacking with Trp2239 (and a more fluctuating interaction with Tyr2225), as well as a significant number of additional residues providing key electrostatic interactions [18]. In the case of oleacein mimetics, it was evident that Trp2239, Tyr2225, and Phe2358 played a central role in the stabilization of their respective complexes with mTOR (Supplementary Table 6).

**Figure 3:**
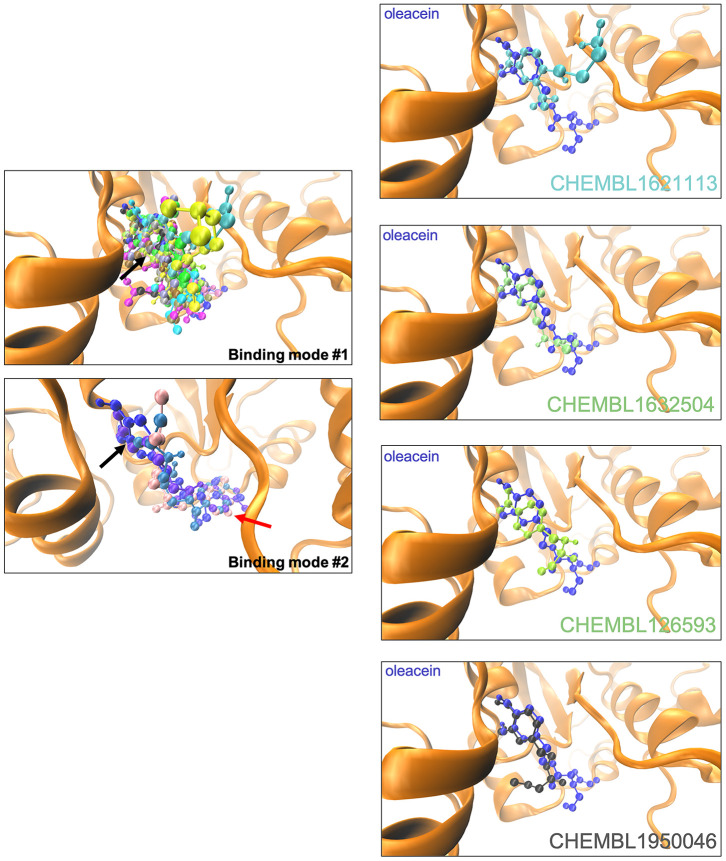
**Binding modes of oleacein mimetics to DNMT.** *Left panels.* Graphical representation of the binding modes of the computationally-predicted oleacein mimetics to the catalytic site of DNMT. The black and red arrows indicate the location of the aromatic rings in the binding modes #1 and #2, respectively. *Right panels.* Graphical representation of the binding modes of parental oleacein and selected oleacein mimetics with anti-CSC activity (Figures 4 and 5) to the catalytic cavity of DNMT.

The binding mode of oleacein to DNMT was predicted to closely resemble that of DNMT inhibitors such as 5-azacytidine, SGI-110, and curcumin [18]. In the case of oleacein mimetics, we were able to predict two different binding modes (Figure 3): one of them shared the oleacein pattern of spatial orientation and included 17 compounds and another one involved only 3 molecules (Figure 3). Rigid docking calculations and MD simulations predicted that the main residues involved in the stabilization of the oleacein-DNMT complex were Ser1446, Pro1125, Asp1143, Phe1145, Gly1150, Leu1151, Asn1158, Val1580, and Gly1223, along with a significant number of additional residues providing key electrostatic interactions. In the case of oleacein mimetics, Phe1145, Trp1170, Pro1224, and Pro1225 were predicted as the main catalytic residues (Supplementary Table 7).

### Oleacein mimetics specifically suppress CSC-driven mammosphere formation

**Figure 4:**
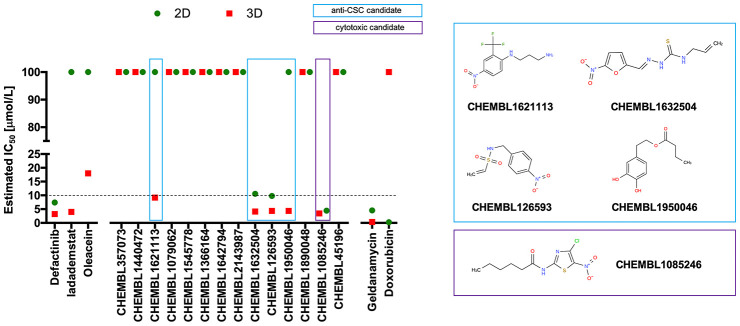
**Phenotypic screening of the anti-CSC activity of oleacein mimetics (I).** *Left.* Comparative analysis of IC_50_ values of the computationally-predicted oleacein mimetics in 2D monolayer cultures and 3D mammosphere systems. With 10 μmol/L as a cutoff, 4/16 compounds tested were more potent in 3D than in 2D and were selected as anti-CSC candidates; 1/16 compounds tested was equally potent in 3D and in 2D and was designated as cytotoxic. *Right*. CHEMBL structures of the computationally-predicted oleacein mimetics with anti-CSC (blue box) and cytotoxic (red box) activity.

To explicitly test the oleacein mimetics on CSC, we measured their effect on *in vitro* tumorsphere formation in low-density non-adherent serum-free medium supplemented with growth factors [29–35], considered one of the gold standards for evaluating CSC self-renewal activity. As a source of CSC, we used the CSC-enriched triple-negative breast cancer model MDA-MB-436, which can form smooth and round tumorspheres (mammospheres) in suspension culture [33]. The Cell2Sphere™ assay [18, 36, 37] was used to evaluate the differential ability of oleacein mimetics to specifically suppress the ability of CSC to survive and proliferate as floating 3D microtumors without promoting nonspecific, cytotoxic effects on the same cells grown in 2D adherent, differentiating conditions (Figure 4).

**Figure 5:**
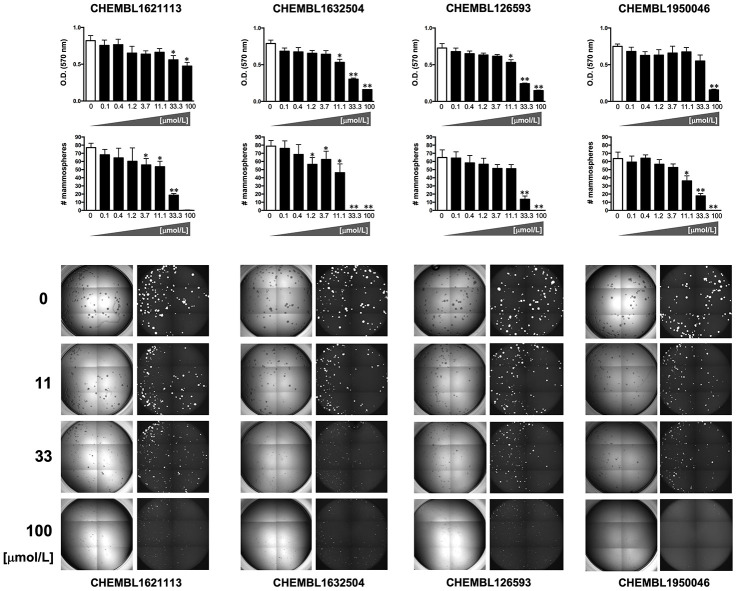
**Phenotypic screening of the anti-CSC activity of oleacein mimetics (II).** *Top panels.* MTT reduction-based measurement of cell viability is expressed as percentage uptake (OD_570_) relative to untreated controls (=100% cell viability). *Bottom panels.* Representative microscope images (×2.5 magnification) of mammospheres formed by MDA-MB-436 cells growing in sphere medium for 6 days in the absence or presence of graded concentrations of oleacein mimetics. The number of mammospheres (>100 μm diameter) is expressed as means (*columns*) ± SD (*bars*). *P < 0.05 and **P < 0.005, statistically significant differences from the untreated (control) group.

Using the focal adhesion kinase inhibitor VS-6063 (defactinib) [38–40] and the lysine-specific demethylase KDM1A inhibitor ORY-1001 (iadademstat) [37, 41] as mechanistically distinct anti-CSC compounds and selecting a 10 μmol/L cut-off for 2D cytotoxicity (i.e., lower than the original IC_50_ value of oleacein [18 ± 5 μmol/L] against CSC-driven mammosphere formation), 4 out of the 14 oleacein mimetics tested specifically suppressed mammosphere formation, namely CHEMBL1621113 (N’-[4-nitro-2-(trifluoromethyl)phenyl]propane-1,3-diamine), CHEMBL1632504 (*(E)-N*-allyl-2-((5-nitrofuran-2-yl)methylene)hydrazinecarbothioamide), CHEMBL126593 (*N*-(4-nitrobenzyl)ethenesulfonamide), and CHEMBL1950046 (3,4-dihydroxyphenethyl butyrate), while not exerting significant cytotoxic effects against differentiated cancer cells growing in 2D in the same low micromolar range (Figure 5). CHEMBL1085246 (*N*-(4-chloro-5-nitrothiazol-2-yl)hexanamide) exhibited anti-CSC activity due to unspecific cytotoxicity against CSC and non-CSC cells (Supplementary Figure 2).

### Oleacein mimetics target ALDH^+^ breast cancer stem cells

**Figure 6:**
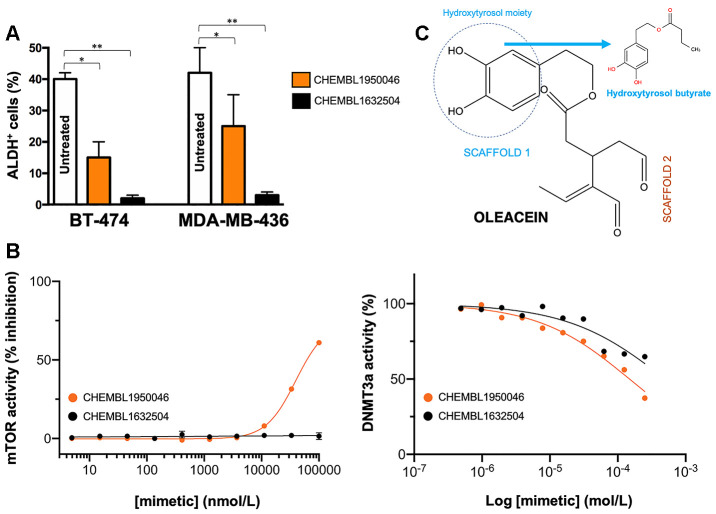
**Phenotypic screening of the anti-CSC activity of oleacein mimetics (III).** (**A**) Changes in the number of ALDH^+^ cells in BT-474 and MDA-MB-436 populations cultured in the absence or presence of 11.1 μmol/L of CHEMBL1950046 and CHEMBL1632504. The results are expressed as percentages means (*columns*) ± SD (*bars*). *P < 0.05 and **P < 0.005, statistically significant differences from the untreated (control) group. (**B**) *Left.* A dose-response inhibition curve of ATP-dependent activity of mTOR kinase was created by plotting FRET signal of the Z´-LYTE Kinase assay as the function of CHEMBL1950046 and CHEMBL1632504 concentrations. *Right.* Dose-response curves of SAM-dependent methylation activity of DNMT3A were created by plotting radioisotope signals of the HotSpot^SM^ assay as the function of CHEMBL1950046 and CHEMBL1632504 concentrations. (**C**) Molecular scaffolds of oleacein.

Oleacein selectively suppresses functional traits of CSC such as the expression of aldehyde dehydrogenase (ALDH) [18], a well-recognized marker of tumorigenic cell fractions enriched for proliferating, epithelial-like CSC capable of self-renewal [31, 32, 35, 42]. We next selected the 2 oleacein mimetics with the best CSC-targeted profile (i.e., anti-CSC activity at low micromolar range and lack of cytotoxic activity against differentiated cancer cells), namely CHEMBL1950046 (3,4-dihydroxyphenethyl butyrate; a.k.a. hydroxytyrosol butyrate) and CHEMBL1632504 (*(E)-N*-allyl-2-((5-nitrofuran-2-yl)methylene)hydrazinecarbothioamide), to evaluate their capacity to target epithelial-like CSC cells with high levels of ALDH1 (ALDH1^+^). To do this, we used the Aldefluor^®^ reagent, which quantifies ALDH activity by measuring the conversion of the ALDH substrate BODIPY aminoacetaldehyde to the fluorescent product BODIPY aminoacetate (Figure 6A). Using HER2-overexpressing BT-474 cells as a breast cancer model naturally enriched with ALDH1^+^ cells, we detected a significant decrease (up to 63% reduction) in the number of ALDH1^+^ cells when BT-474 cells were treated with a non-cytotoxic concentration (10 μmol/L) of CHEMBL1950046 (hydroxytyrosol butyrate). A more pronounced effect was seen with CHEMBL1632504 (*(E)-N*-allyl-2-((5-nitrofuran-2-yl)methylene)hydrazinecarbothioamide), which significantly decreased the proportion of ALDH^+^ cells from 40±2% in untreated BT-474 cells to levels as low as 2±1% (96% reduction). To corroborate the ability of oleacein mimetics to target ALDH1^+^ epithelial-like CSC irrespective of the mutational landscape of cancer cells, we employed triple-negative MDA-MB-436 cells as a second breast cancer model naturally enriched with ALDH1^+^ cells. Treatment with hydroxytyrosol butyrate decreased the ALDH1^+^ cell content of MDA-MB-436 by approximately 40%. Remarkably, the large population of ALDH1^+^ cells in untreated MDA-MB-436 cultures (42±8%) was drastically reduced by 93% (from 42±8% to 3±1%) in the presence of *(E)-N*-allyl-2-((5-nitrofuran-2-yl)methylene)hydrazinecarbothioamide.

Preservation of the oleacein binding mode is required for a dual mTOR/DNMT inhibitory activity but not for their anti-CSC behavior of oleacein mimetics. We finally evaluated whether the selected mimetics hydroxytyrosol butyrate and *(E)-N*-allyl-2-((5-nitrofuran-2-yl)methylene)hydrazinecarbothioamide preserved the dual anti-mTOR/DNMTactivity of the parental oleacein.

We first employed the FRET-based Z-LYTE™ Kinase Assay to test the ability of the selected oleacein mimetics to inhibit mTOR activity. Ten concentrations of hydroxytyrosol butyrate and *(E)-N*-allyl-2-((5-nitrofuran-2-yl)methylene)hydrazinecarbothioamide spanning over five logarithmic decades were selected. Figure 6B shows the mTOR activity rate as a function of oleacein mimetics concentration. Hydroxytyrosol butyrate inhibited mTOR activity with an IC_50_ of ~39 μmol/L; *(E)-N*-allyl-2-((5-nitrofuran-2-yl)methylene)hydrazinecarbothioamide was unable to decrease mTOR activity even at the maximum concentration tested.

We finally carried out a radioisotope-based methyltransferase profiling measuring the DNMT3A-catalyzed incorporation of S-adenosyl-L[methyl-^3^H]methionine (SAM[^3^H]) into DNA (DNA 5-[methyl-^3^H]-cytosine) in the absence or presence of oleacein mimetics. The selected oleacein mimetics were tested in 10-dose IC_50_ mode with 2-fold serial dilution and reactions were carried out at 1 μmol/L SAM. Although hydroxytyrosol butyrate decreased DNMT3A activity in a dose-dependent manner, concentrations higher than 150 μmol/L were necessary to reach the IC_50_ value. *(E)-N*-allyl-2-((5-nitrofuran-2-yl)methylene)hydrazinecarbothioamide did not reach the half maximal inhibitory concentration of DNMT3a activity even at the highest concentration tested.

## DISCUSSION

The molecular frameworks of natural products can provide feasible and innovative templates for medicinal chemistry and drug discovery [43]. But, despite the long tradition of natural product-inspired discovery of synthetic compounds, there has been little effort to utilize EVOO biophenols chemotypes as a springboard for lead discovery. Here, we carried out such a drug discovery approach to uncover new compounds capable of phenotypically mimicking the anti-CSC effects of the EVOO dihydroxy-phenol oleacein.

We took advantage of modern bioinformatics approaches with the aim of identifying physicochemical mimetics of the anti-CSC behavior of EVOO-derived oleacein. First, the somewhat structurally complex framework of the dialdehydic form of decarboxymethyl elenolic acid linked to hydroxytyrosol (i.e., oleacein) was computationally captured in terms of molecules with oleacein-like physico-chemical profiles. Second, we *in silico* compared the binding modes of the top 20 computationally-predicted oleacein mimetics to the two molecular targets originally involved in the capacity of oleacein to specifically suppress the functional traits of tumor-initiating CSC (i.e., mTOR and DNMT) [14]. Third, we phenotypically explored the computationally-discovered oleacein biomimetics in terms of their anti-CSC activity. Fourth, we evaluated the structure-mTOR/DNMT bioactivity relationship of the most promising oleacein-mimetic candidates. By doing so, four oleacein mimetics, namely N’-[4-nitro-2-(trifluoromethyl)phenyl]propane-1,3-diamine, *(E)-N*-allyl-2-((5-nitrofuran-2-yl)methylene)hydrazinecarbothioamide, *N*-(4-nitrobenzyl)ethenesulfonamide, and 3,4-dihydroxyphenethyl butyrate (a.k.a. hydroxytyrosol butyrate), fulfilled the first phenotypic endpoint of the selection criteria, which was the specific suppression of the 3D mammosphere forming capability of CSC in the low micromolar range without highly significant cytotoxic effects against differentiated cancer cells growing in 2D cultures in the same range of concentrations. Moreover, non-cytotoxic concentrations of the oleacein mimetics hydroxytyrosol butyrate and *(E)-N*-allyl-2-((5-nitrofuran-2-yl)methylene)hydrazinecarbothioamide efficiently suppressed the population of ALDH1^+^ epithelial-like proliferating CSC [31, 32, 35, 42], a second phenotypic endpoint of the selection criteria for anti-CSC candidates.

The fact that the oleacein mimetics-responsive phenotypes were exclusively manifested under 3D stem cell culture conditions along with their capacity to specifically and potently suppress (>90%) ALDH1^+^ CSC-like cellular states irrespective of the mutational landscape of the cancer cell population strongly suggested that their mechanism of action targets the biological functioning of cancer stemness *per se*. Hydroxytyrosol butyrate is a chemically-modified (alkyl ester) lipophilic version of hydroxytyrosol that is more stable than parental hydroxytyrosol under biological conditions [44–49]. The fact that the inclusion of a short-medium lipophilic chain in the hydroxytyrosol molecule sufficed to recapitulate, at least in part, both the anti-CSC behavior and the anti-mTOR/DNMT inhibitory activity of the parental oleacein highlights the functional relevance of the dihydroxybenzene moiety within the phenolic part of oleacein, a scaffold that seems to be a crucial mediator of the metabolo-epigenetic modulatory effects of oleacein (e.g., COMT, IDH1, LSD1 [18, 22, 50–52]) *via* formation of stacking interactions, coordination with metal ions, and/or establishment of hydrophobic and/or hydrogen bond interactions through the hydroxyl groups or the aromatic ring (Figure 6C). The second oleacein scaffold, which comprises the secoiridoid dialdehyde part, might be involved in the stabilization of oleacein *via* hydrophobic interactions within the binding pocket of the targeted proteins. Accordingly, although hydroxytyrosol butyrate preserved the original double occupancy of oleacein within the catalytic sites of mTOR and DNMT, the sole dihydroxybenzene moiety does not suffice to fully preserve the low-micromolar biological activity of oleacein against mTOR and DNMT enzymatic activities. *(E)-N*-allyl-2-((5-nitrofuran-2-yl)methylene)hydrazinecarbothioamide, originally described as an inhibitor of the *Trypanosoma cruzi* triosephosphate isomerase [53], lacked the original binding sites of oleacein to mTOR and DNMT, thereby fully losing the original ability of oleacein to operate as a dual mTOR/DNMT inhibitor. *(E)-N*-allyl-2-((5-nitrofuran-2-yl)methylene)hydrazinecarbothioamide, however, appeared to operate as an optimized mimetic of oleacein capable of exhibiting a very promising and potent activity against ALDH1-positive breast CSC. These findings can be consistent with the notion that preservation of the original binding mode of oleacein to mTOR and DNMT is an obligatory requirement for a dual mTOR/DNMT inhibitory activity of hydroxytyrosol-related oleacein mimetics (e.g., hydroxytyrosol butyrate) with anti-CSC activity; for hydroxytyrosol-unrelated oleacein mimetics (e.g., *(E)-N*-allyl-2-((5-nitrofuran-2-yl)methylene)hydrazinecarbothioamide), however, the absence of a dual mTOR/DNMT inhibitory activity is dispensable for an efficient suppression of the ALDH^+^-CSC functional phenotype.

We provide, to the best of our knowledge, the first evidence that the pharma-nutritional properties of oleacein that elicit its functioning as an anti-CSC compound can be phenocopied through the use of mimetics that capture its physico-chemical properties. Although we acknowledge that further studies are needed to validate the ability of oleacein mimetics to functionally deplete tumor-initiating CSC-like states *in vivo* and the mechanisms underlying their mode of action, it is reasonable to suggest that a biomimicry design process might guide the development of synthetically tractable small molecules capable of phenotypically imitating the anti-CSC chemistry of complex EVOO phenolics such as oleacein.

## MATERIALS AND METHODS

### Preparation and analytical characterization of oleacein mimetics

### CHEMBL2143987 (N-(2-(Dimethylamino)ethyl)-2-(4-nitrophenyl)acetamide)

A mixture of 4-nitrophenylacetic acid (100 mg, 0.552 mmol) and CDI (94mg, 0.58 mmol) in DMF (1.4 mL) was stirred at 50° C for 10 min. The solution was cooled to 20° C, *N,N*-dimethylaminoethylamine (63.6 μL, 0.58 mmol) was added dropwise and the solution stirred for 2 h. The solution was poured into water and extracted with EtOAc (3×). The combined organic extracts were washed with water, brine, dried, and the solvent removed under reduced pressure. The residue was chromatographed, eluting with a DCM/MeOH (1%NH3) yielding *N*-(*N*,*N*-dimethylaminoethyl)-2-4-nitrophenylacetamide (27 mg, 19.5%).

### CHEMBL1632504 ((E)-N-Allyl-2-((5-nitrofuran-2-yl)methylene)hydrazinecarbothioamide)

5-Nitrofuran-2-carbaldehyde (100 mg, 0.709 mmol), *N*-allylhydrazinecarbothioamide (93 mg, 0.709 mmol), *p*-TSA (6.74 mg, 0.035 mmol) and toluene (7.0 mL) were stirred at room temperature until the aldehyde was not present (1.5h). The solid formed (136 mg, 75%) was collected by filtration.

### CHEMBL126593 (N-(4-Nitrobenzyl)ethenesulfonamide)

4-Nitrophenyl)methanamine (100 mg, 0.657 mmol) was dissolved in DCM (620 μL, dry) at 0° C with stirring under N2 to which a 4-methylmorpholine (145 μl, 1.314 mmol) was added with stirring. A solution of y 2-chloroethanesulfonyl chloride (68.7 μl, 0.657 mmol) dissolved in DCM (620 μL, dry) was added at 0° C with stirring 10 min under N_2_, after which time the reaction mixture was stirred at room temperature overnight. The reaction mixture was extracted with dilute hydrochloric acid and the organic layers were collected, dried (MgSO_4_), filtered and the solvent removed under reduced pressure. The crude product was purified by column chromatography (EtOAc/n-hexane 1/2). The product was obtained as a white solid (11 mg, 7%).

### CHEMBL1950046 (3,4-Dihydroxyphenethyl butyrate)

Lipase P (25 mg) and vinyl butyrate (412 μl, 3.24 mmol) were added to a solution of 4-(2-hydroxyethyl)benzene-1,2-diol (25 mg, 0.162 mmol) in *t*BuOMe (Volume: 5792 μl) and the mixture was shaken at 40° C for 60 min. The reaction was quenched by filtering off enzyme and the filtrate was evaporated *in vacuo*. The resulting residue was dissolved in EtOAc and washed with sat. NaHCO_3_ and brine then dried (MgSO_4_) followed by filtration and evaporation to dryness. 32 mg (89%) of compound identified as the title compound were obtained.

### CHEMBL1890048 (2-Methoxy-N-(2-methyl-5-nitrophenyl)acetamide)

To a solution of 2-methyl-5-nitroaniline (100 mg, 0.657 mmol) in DCM (0.04 M), TEA (0.137 ml, 0.986 mmol) and2-methoxyacetyl chloride (0.066 μl, 0.723 mmol) were added. The reaction mixture was stirred at room temperature for 4 h. 103 mg (70%) of compound identified as the title compound were obtained.

### CHEMBL1085246 (N-(4-Chloro-5-nitrothiazol-2-yl)hexanamide)

Hexanoyl chloride (38.2 μl, 0.278 mmol) was dissolved in THF (0.1 M) and cooled to -78° C then 4-chloro-5-nitrothiazol-2-amine (50 mg, 0.278 mmol) was added in one portion. DIPEA (1.1 eq) was added to the resulting slurry at -78° C and the solution was held at this temperature for 10 min then allowed to warm to room temperature overnight. The solution was diluted with EtOAc and washed with sat. NaHCO_3_, 1M HCl and brine then dried (MgSO_4_) followed by filtration and evaporation to dryness. The resulting residue was purified by gradient flash column chromatography (10-60% EtOAc/hexanes or 1-2% MeOH/CH_2_Cl_2_) to obtain 22 mg (28.5%) of compound identified as the title compound.

### CHEMBL45196 (4-((5-Chloro-2-nitrophenyl)amino)-4-oxo-2-(2,2,2-trifluoroacetamido)butanoic acid)

A mixture of 5-chloro-2-nitroaniline (50 mg, 0.290 mmol) and (*S*)-*N*-(2,5-dioxotetrahydrofuran-3-yl)-2,2,2-trifluoroacetamide (61.2 mg, 0.290 mmol) was irradiated for 60 minutes in a microwave (130° C, 200 psi, 200W). The residue was purified by reversed-phase flash chromatography, yielding 14 mg (12%) of compound identified as the title compound.

CHEMBL357073 (6-[(4-nitrophenyl)formamido]hexanoic acid), CHEMBL1545778 ([2-(methylcarbamoylamino)-2-oxo-ethyl] (E)-3-(3-bromophenyl)prop-2-enoate), CHEMBL1366164 (ethyl 2-[(2-methyl-5-nitro-phenyl)amino]-2-oxoacetate), and CHEMBL1642794 ([2-(tert-butylamino)-2-oxo-ethyl] 4-nitrobenzoate) were purchased from Enamine (EN300-302808, Z18646098, EN300-236023, and Z19756482, respectively; Kiev, Ukraine). CHEMBL1440472 (2- [(6- chloro- 3- nitro- 2-pyridinyl)amino]-3-methylbutanoic acid) was purchased from Key Organics (MS-1625; Bedford, MA). CHEMBL1621113 (N-[4-nitro-2-(trifluoromethyl)phenyl]propane-1,3-diamine) and CHEMBL1079062 ((Z)-4-[(4-nitrophenyl)amino]-4-oxobut-2-enoic acid) were purchased from ABCR GmbH (AB141160 and AB414326, respectively; Karlsruhe, Germany).

### Analytical and spectroscopic characterization of oleacein mimetics

### NMR

NMR spectra were recorded on an Agilent VNMRS-400 (^1^H at 400.10 MHz). *HPLC-MS.* HPLC-MS were performed with a High-Performance Liquid Chromatography Thermo Ultimate 3000SD (Thermo Scientific Dionex) coupled to a photodiode array detector and a mass spectrometer LTQ XL ESI-ion trap (Thermo Scientific); 5μl of sample MeOH were injected (c=0.5mg/mL). Data from mass spectra were analyzed by electrospray ionization in positive and negative mode and peaks are given m/z (% of basis peak). The mobile phase used was a mixture of A = water + 0.05 formic acid and B = Acetonitrile + 0.05 formic acid with method described as follows: flow 0.5 mL/min; 5% B for 0.5 min; 5%-100% B in 5 min, 100% B for 2min.

### Virtual screening

Virtual profiling was performed with ligand- and structure-based software tools, using the chemical structure of oleacein as a seed, as described [54]. Briefly, the 3D virtual profiling tool compares a query molecule (i.e., oleacein) with the structures present in the Chembl(v19) reference database using Comparative Molecular Similarity Indices Analysis (CoMSIA) fields on a 3D grid. Molecules were compared according to their relationship with their environment using the 3D descriptors topologic surface area, lipophilicity, hydrogen bond donors/acceptors count, and Van der Waals radii, among others, thereby obtaining biomimetic compounds with different structures.

### Docking and molecular dynamics calculations

All docking, MD calculations and MM/GBSA rescoring were carried out as described [18, 22, 54].

### Cell viability

Cell viability was determined using a standard colorimetric MTT-based reduction assay 72 h after exposure to graded concentrations of oleacein mimetics.

### Mammosphere formation

Mammosphere formation was monitored using Cell2Sphere™ assays (StemTek Therapeutics, Bilbao, Spain). Graded concentrations of oleacein mimetics were added to triplicate sets of wells on day 1 and the number of 6-day-old mammospheres was recorded as a measurement of CSC content. Images were recorded using a BioTek Cytation 5 image cytometer at 2.5× magnification. Prior to image acquisition, spheroid cultures were stained with a fluorescent vital dye to increase the accuracy of spheroid detection and analysis. The system was then set to count number, size, and aspect ratio of the objects. Thresholds were set to >100 μm in size and 0.4 as aspect ratio (with 1 being the aspect ratio of a perfect circle).

### Aldefluor activity assay

The ALDEFLUOR^®^ assay (StemCell Technologies, Vancouver, BC, Canada) was performed with or without the addition of hydroxytyrosol butyrate and *(E)-N*-allyl-2-((5-nitrofuran-2-yl)methylene)hydrazinecarbothioamide for 48 h.

### mTOR and DNMT activity/inhibition assays

IC_50_ determinations for FRAP1 (mTOR) of oleacein mimetics were outsourced to Invitrogen (Life Technologies) using the FRET-based Z-LYTE™ SelectScreen Kinase Profiling Service. The effect of oleacein mimetics on the enzymatic activities of the recombinant human DNMT3A was outsourced to Reaction Biology Corp. (Malvern, PA) using HotSpot^SM^, a nanoliter-scale radioisotope filter binding platform.

**Statistical analysis**

All statistical analyses were performed using GraphPad Prism software (San Diego, CA). Data are presented as mean ± S.D. Comparisons of means of ≥ 3 groups were performed by analysis of variance (ANOVA) and the existence of individual differences, in case of significant *F* values at ANOVA, were assessed by multiple contrasts. *P* values < 0.05 and <0.005 were considered to be statistically significant (denoted as * and **, respectively). All statistical tests were two-sided.

## Supplementary Material

Supplementary Figures

Supplementary Tables 1, 2, 3, 4 and 5

Supplementary Table 6

Supplementary Table 7
