# Supplementary material for: Mimetics of extra virgin olive oil phenols with anti-cancer stem cell activity
Source: Aging (Albany NY). 2020 Nov 9;12(21):21057–75. doi: 10.18632/aging.202154 (PMC7695371; doi:10.18632/aging.202154)
Supplement: Supplementary Figures [file aging-12-202154-s001..pdf]

SUPPLEMENTARY FIGURES

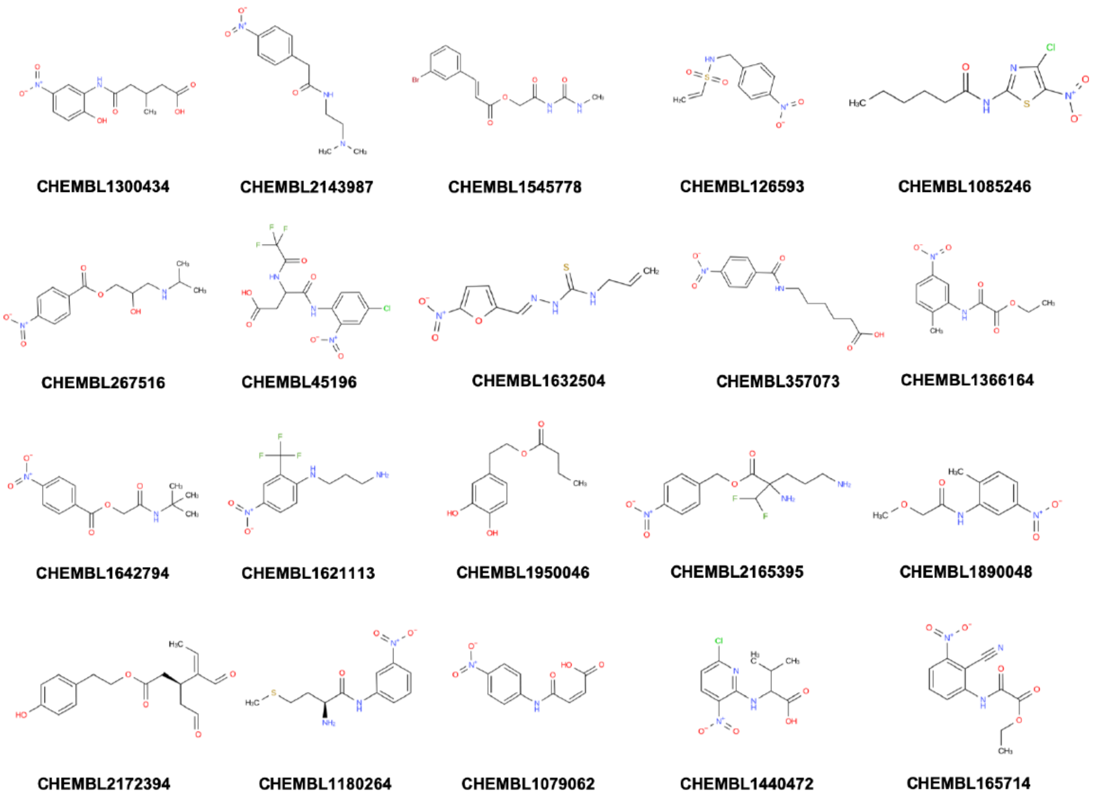

Supplementary Figure 1. CHEMBL structures of the computationally-predicted oleacein mimetics

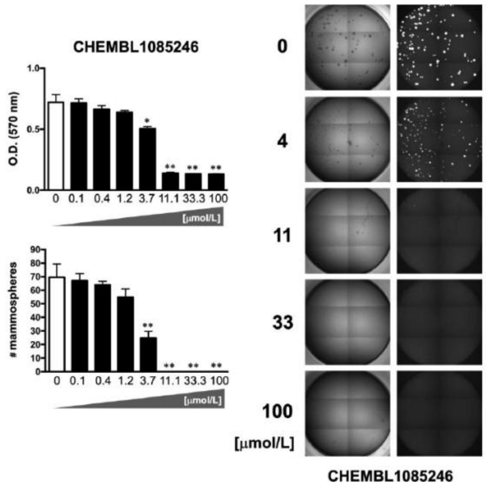

Supplementary Figure 2. ***Top panels.*** **MTT reduction-based measurement of cell viability is expressed as percentage uptake (OD_{570}) relative to untreated controls (=100% cell viability).** *Bottom panels.* Representative microscope images (×2.5 magnification) of mammospheres formed by MDA-MB-436 cells growing in sphere medium for 6 days in the absence or presence of graded concentrations of CHEMBL1085246. The number of mammospheres (>100 µm diameter) is expressed as means (*columns*) ± SD (*bars*). **P* < 0.05 and ***P* < 0.005, statistically significant differences from the untreated (control) group.
