# Supplementary material for: Mimetics of extra virgin olive oil phenols with anti-cancer stem cell activity
Source: Aging (Albany NY). 2020 Nov 9;12(21):21057–75. doi: 10.18632/aging.202154 (PMC7695371; doi:10.18632/aging.202154)
Supplement: Supplementary Tables 1, 2, 3, 4 and 5 [file aging-12-202154-s002..pdf]

SUPPLEMENTARY TABLES

Supplementary Table 1. Docking binding energies and MM/GBSA-based energy rescoring calculations of oleacein against mTOR and DNMT.

Cavity	Docking ΔG kcal/mol	MM/GBSA ΔG kcal/mol	Target / PDBID
**Crystallographic cavity**	-7.1/-7.1	-26.8226	mTOR / 4JT6
**Cavity1**	-7.3/-6.9	-17.155	mTOR / 4JT6
**Cavity4**	-7.6/-7.8	-36.9931	mTOR / 4JT6
**Cavity8**	-7.4/-7.3	-21.8981	mTOR / 4JT6
**Crystallographic cavity**	-7.9/-7.6	-30.567	DNMT / 4WXX
**Cavity1**	-7.7/-7.7	-25.2792	DNMT / 4WXX
**Cavity2**	-7.2/-7.2	-36.5163	DNMT / 4WXX
**Cavity3**	-7.3/-7.3	-34.0772	DNMT / 4WXX

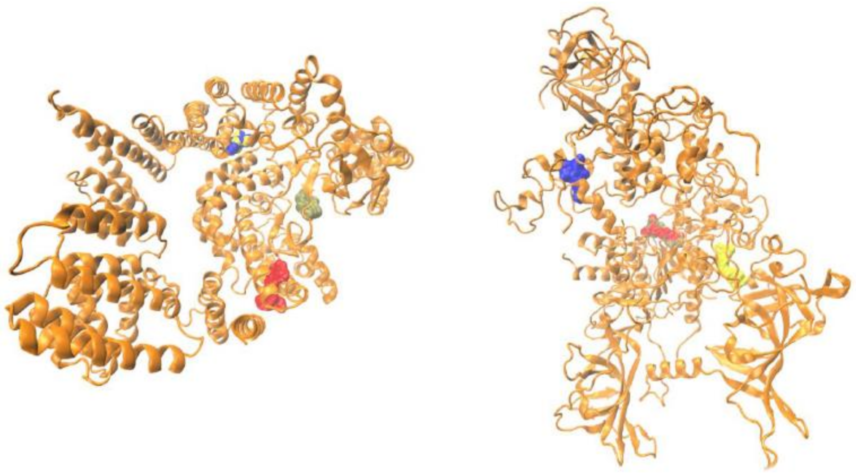

Graphical representation of parental oleacein bound to several cavities of mTOR (4JT6, *left*) and DNMT (4WXX, *right*). Oleacein is colored in gold. In the case of mTOR, oleacein poses at cavities 1, 4, and 8, colored in blue, red, and yellow, respectively. In the case of DNMT, oleacein poses at cavities 1, 2, and 3, colored in blue, red, and yellow, respectively.

Supplementary Table 2. Docking binding energies of oleacein mimetics against the crystallographic cavities of mTOR and DNMT.

Oleacein mimetic	Target/ PDBID	ΔG kcal/mol R0 / R1	Target / PDBID	ΔG kcal/mol R0 / R1
CHEMBL2172394	mTOR / 4JT6	-6.6 / -6.9	DNMT / 4WXX	-6.9 / -7.1
CHEMBL1085246	mTOR / 4JT6	-6.5 / -6.7	DNMT / 4WXX	-7.6 / -7.5
CHEMBL357073	mTOR / 4JT6	-7.3 / -7.2	DNMT / 4WXX	-7.8 / -7.6
CHEMBL1632504	mTOR / 4JT6	-5.9 / -6.0	DNMT / 4WXX	-7.3 / -7.2
CHEMBL126593	mTOR / 4JT6	-6.7 / -6.8	DNMT / 4WXX	-6.8 / -6.8
CHEMBL1950046	mTOR / 4JT6	-6.7 / -6.7	DNMT / 4WXX	-6.7 / -6.7
CHEMBL1440472	mTOR / 4JT6	-6.1 / -6.1	DNMT / 4WXX	-7.0 / -7.0
CHEMBL1300434	mTOR / 4JT6	-6.6 / -6.6	DNMT / 4WXX	-7.5 / -7.5
CHEMBL1890048	mTOR / 4JT6	-6.3 / -6.4	DNMT / 4WXX	-6.8 / -6.8
CHEMBL1180264	mTOR / 4JT6	-6.1 / -6.1	DNMT / 4WXX	-7.3 / -7.4
CHEMBL165714	mTOR / 4JT6	-6.3 / -6.3	DNMT / 4WXX	-7.3 / -7.3
CHEMBL1621113	mTOR / 4JT6	-6.7 / -6.6	DNMT / 4WXX	-7.0 / -7.2
CHEMBL1079062	mTOR / 4JT6	-7.2 / -7.2	DNMT / 4WXX	-7.7 / -7.7

CHEMBL267516	mTOR / 4JT6	-6.6 / -6.7	DNMT / 4WXX	-7.3 / -7.1
CHEMBL154778	mTOR / 4JT6	-8.8 / -8.6	DNMT / 4WXX	-8.1 / -8.1
CHEMBL1366164	mTOR / 4JT6	-6.9 / -6.9	DNMT / 4WXX	-7.4 / -7.4
CHEMBL1642794	mTOR / 4JT6	-7.0/ -7.0	DNMT / 4WXX	-7.6 / -7.6
CHEMBL2165395	mTOR / 4JT6	-6.5 / -6.4	DNMT / 4WXX	-7.3 / -7.3
CHEMBL45196	mTOR / 4JT6	-7.1 / -6.6	DNMT / 4WXX	-8.3 / -8.3
CHEMBL2143987	mTOR / 4JT6	-6.2 / -6.2	DNMT / 4WXX	-6.9 / -7.0

Each calculation was performed twice (R0, R1) to avoid false positives. Differences less than 1 kcal/mol are negligible.

Supplementary Table 3. Docking binding energies of oleacein mimetics against the best cavity of mTOR and DNMT shared with oleacein.

Oleacein mimetic	Cavity	Target / PDBID	ΔG kcal/mol R0 / R1	Cavity	Target / PDBID	ΔG kcal/mol R0 / R1
CHEMBL2172394	Cavity4	mTOR / 4JT6	-7.8 / -7.8	Cavity3	DNMT / 4WXX	-7.3 / -7.4
CHEMBL1085246	Cavity4	mTOR / 4JT6	-7.6 / -7.4	Cavity2	DNMT / 4WXX	-7.0 / -7.0
CHEMBL357073	Cavity4	mTOR / 4JT6	-7.7 / -7.6	Cavity2	DNMT / 4WXX	-7.4 / -7.6
CHEMBL1632504	Cavity8	mTOR / 4JT6	-6.8 / -6.7	Cavity2	DNMT / 4WXX	-7.2 / -7.0
CHEMBL126593	Cavity1	mTOR / 4JT6	-7.1 / -7.1	Cavity2	DNMT / 4WXX	-6.7 / -6.8
CHEMBL1950046	Cavity4	mTOR / 4JT6	-7.1 /-7.1	Cavity2	DNMT / 4WXX	-6.8 / -6.8
CHEMBL1440472	Cavity8	mTOR / 4JT6	-7.0 / -7.4	Cavity4	DNMT / 4WXX	-7.0 / -7.0
CHEMBL1300434	Cavity8	mTOR / 4JT6	-7.2 / -7.2	Cavity2	DNMT / 4WXX	-7.7 / -7.8
CHEMBL1890048	Cavity4	mTOR / 4JT6	-7.0 / -7.0	Cavity2	DNMT / 4WXX	-6.9 / -6.8
CHEMBL1180264	Cavity8	mTOR / 4JT6	-7.1 / -6.7	Cavity2	DNMT / 4WXX	-7.3 / -7.4
CHEMBL165714	Cavity8	mTOR / 4JT6	-7.2 / -7.4	Cavity1	DNMT / 4WXX	-6.9 / -6.8
CHEMBL1621113	Cavity1	mTOR / 4JT6	-7.0 / -6.5	Cavity2	DNMT / 4WXX	-7.3 / -7.3
CHEMBL1079062	Cavity8	mTOR / 4JT6	-7.3 / -7.5	Cavity2	DNMT / 4WXX	-7.5 / -7.9
CHEMBL267516	Cavity8	mTOR / 4JT6	-7.1 / -7.1	Cavity2	DNMT / 4WXX	-7.2 / -7.1
CHEMBL154778	Cavity4	mTOR / 4JT6	-8.1 / -8.2	Cavity2	DNMT / 4WXX	-8.0 / -7.8
CHEMBL1366164	Cavity4	mTOR / 4JT6	-7.2 / -7.4	Cavity2	DNMT / 4WXX	-7.2 / -7.2
CHEMBL1642794	Cavity1	mTOR / 4JT6	-7.8 / -7.6	Cavity2	DNMT / 4WXX	-7.5 / -7.5
CHEMBL2165395	Cavity8	mTOR / 4JT6	-7.6 / -7.6	Cavity2	DNMT / 4WXX	-7.4 / -7.0
CHEMBL45196	Cavity4	mTOR / 4JT6	-8.4 / -8.0	Cavity1	DNMT / 4WXX	-8.3 / -8.1
CHEMBL2143987	Cavity4	mTOR / 4JT6	-7.1 / -6.8	Cavity3	DNMT / 4WXX	-6.9 / -7.0

Each calculation was performed twice (R0, R1) to avoid false positives. Differences less than 1 kcal/mol are negligible.

Supplementary Table 4. MM/GBSA-based binding energy rescoring calculations over MD simulations of computationally-predicted oleacein mimetics against the crystallographic cavities of mTOR and DNMT.

Oleacein mimetic	Target/ PDBID	ΔG kcal/mol	Target / PDBID	ΔG kcal/mol
CHEMBL2172394	mTOR / 4JT6	-18.4177	DNMT / 4WXX	-11.8887
CHEMBL1085246	mTOR / 4JT6	-27.4436	DNMT / 4WXX	-17.8140
CHEMBL357073	mTOR / 4JT6	-25.0102	DNMT / 4WXX	-28.4676
CHEMBL1632504	mTOR / 4JT6	-25.7896	DNMT / 4WXX	-38.2609
CHEMBL126593	mTOR / 4JT6	-29.2106	DNMT / 4WXX	-25.7134
CHEMBL1950046	mTOR / 4JT6	-20.2999	DNMT / 4WXX	-24.3167
CHEMBL1440472	mTOR / 4JT6	-16.6468	DNMT / 4WXX	-27.5899
CHEMBL1300434	mTOR / 4JT6	-38.7014	DNMT / 4WXX	-33.3421
CHEMBL1890048	mTOR / 4JT6	-19.6392	DNMT / 4WXX	-26.0912
CHEMBL1180264	mTOR / 4JT6	-18.2272	DNMT / 4WXX	-31.7196
CHEMBL165714	mTOR / 4JT6	-16.1321	DNMT / 4WXX	-12.1247
CHEMBL1621113	mTOR / 4JT6	-22.9663	DNMT / 4WXX	-26.6488

CHEMBL1079062	mTOR / 4JT6	-17.4413	DNMT / 4WXX	-24.2025
CHEMBL267516	mTOR / 4JT6	-27.371	DNMT / 4WXX	-32.8788
CHEMBL154778	mTOR / 4JT6	-30.5493	DNMT / 4WXX	-21.6215
CHEMBL1366164	mTOR / 4JT6	-24.3303	DNMT / 4WXX	-15.4957
CHEMBL1642794	mTOR / 4JT6	-24.1435	DNMT / 4WXX	-16.1264
CHEMBL2165395	mTOR / 4JT6	-19.8235	DNMT / 4WXX	-33.4134
CHEMBL45196	mTOR / 4JT6	-27.2624	DNMT / 4WXX	-24.5175
CHEMBL2143987	mTOR / 4JT6	-32.407	DNMT / 4WXX	-36.4821

Suppleemntary Table 5. MM/GBSA-based binding energy rescoring calculations over MD simulations of computationally-predicted oleacein mimetics against against the best cavity of mTOR and DNMT shared with oleacein.

Oleacein candidate	Cavity	Target / PDBID	ΔG kcal/mol	Cavity	Target / PDBID	ΔG kcal/mol
CHEMBL2172394	Cavity4	mTOR / 4JT6	-34.392	Cavity3	DNMT / 4WXX	-31.0757
CHEMBL1085246	Cavity4	mTOR / 4JT6	-19.6725	Cavity2	DNMT / 4WXX	-36.9931
CHEMBL357073	Cavity4	mTOR / 4JT6	-33.5462	Cavity2	DNMT / 4WXX	-34.3628
CHEMBL1632504	Cavity8	mTOR / 4JT6	-24.6272	Cavity2	DNMT / 4WXX	-36.6319
CHEMBL126593	Cavity1	mTOR / 4JT6	-26.6329	Cavity2	DNMT / 4WXX	-35.3592
CHEMBL1950046	Cavity4	mTOR / 4JT6	-31.6794	Cavity2	DNMT / 4WXX	-21.7283
CHEMBL1440472	Cavity8	mTOR / 4JT6	-21.2853	Cavity4	DNMT / 4WXX	-29.360
CHEMBL1300434	Cavity8	mTOR / 4JT6	-27.361	Cavity2	DNMT / 4WXX	-33.9773
CHEMBL1890048	Cavity4	mTOR / 4JT6	-21.2089	Cavity2	DNMT / 4WXX	-26.2952
CHEMBL1180264	Cavity8	mTOR / 4JT6	-29.4140	Cavity2	DNMT / 4WXX	-32.3981
CHEMBL165714	Cavity8	mTOR / 4JT6	-21.4634	Cavity1	DNMT / 4WXX	-30.3770
CHEMBL1621113	Cavity1	mTOR / 4JT6	-21.0309	Cavity2	DNMT / 4WXX	-29.3269
CHEMBL1079062	Cavity8	mTOR / 4JT6	-24.7585	Cavity2	DNMT / 4WXX	-24.4205
CHEMBL267516	Cavity8	mTOR / 4JT6	-44.6454	Cavity2	DNMT / 4WXX	-28.1508
CHEMBL154778	Cavity4	mTOR / 4JT6	-25.0387	Cavity2	DNMT / 4WXX	-22.9832
CHEMBL1366164	Cavity4	mTOR / 4JT6	-17.8085	Cavity2	DNMT / 4WXX	-19.6201
CHEMBL1642794	Cavity1	mTOR / 4JT6	-19.439	Cavity2	DNMT / 4WXX	-20.6555
CHEMBL2165395	Cavity8	mTOR / 4JT6	-27.2639	Cavity2	DNMT / 4WXX	-25.8227
CHEMBL45196	Cavity4	mTOR / 4JT6	-17,1961	Cavity1	DNMT / 4WXX	-32,1555
CHEMBL2143987	Cavity4	mTOR / 4JT6	-40.3344	Cavity3	DNMT / 4WXX	-43.6863
