# Supplementary material for: Mimetics of extra virgin olive oil phenols with anti-cancer stem cell activity
Source: Aging (Albany NY). 2020 Nov 9;12(21):21057–75. doi: 10.18632/aging.202154 (PMC7695371; doi:10.18632/aging.202154)
Supplement: Supplementary Table 6 [file aging-12-202154-s003..docx]

**Supplementary Table 6.** **Key interacting residues of oleacein mimetics to the catalytic site of mTOR.** Interactions other than electrostatic are highlighted in yellow (possible) or green (reliable).

| Oleacein mimetic | Main catalytic residues | Other relevant residues |
| --- | --- | --- |
| CHEMBL2172394 | Leu2185  Lys2187  Ile2356  Ser2342  Trp2239 (π-π stacking) | Pro2169  Asp2244  Thr2245  Asn2343  Asp2357  Met2345  Gln2167 |
| CHEMBL1085246 | Trp2239 (π-πstacking/cation-π stacking)  Ser2342 | Ile2163  Pro2169  Leu2185  Thr2245  Met2345  Ile2356 |
| CHEMBL357073 | Pro2169  Leu2185  Lys2187  Thr2245 | Ile2163  Leu2192  Ile2337  Trp2239  Ile2356  Asp2357 |
| CHEMBL1632504 | Leu2185  Trp2239 (cation-π stacking)  Val2240  Met2345  Ile2356 | Leu2187  Ile2237  Gly2238  Thr2245 |
| CHEMBL126593 | Trp2239 (π-π stacking)  Val2240  Met2345  Ile2356 | Ile2163  Leu2169  Tyr2225  Leu2354 |
| CHEMBL1950046 | Leu2187  Leu2192  Trp2239 (π-π stacking)  Ile2356  Asp2357 | Leu2195  Tyr2225  Ile2237  Val2240 |
| CHEMBL1440472 | Lys2187  Asn2343  Asp2357 | Pro2169  Leu2185  Leu2192  Tyr2225 (halogen bonding)  Ile2337  Ile2356 |
| CHEMBL1300434 | Lys2187  Leu2192  Asp2195  Ile2356  Asp2357  Phe2358 | Leu2185  Asp2191  Gln2194  Tyr2225  Ile2237  Gly2359 |
| CHEMBL1890048 | Trp2239(π-π stacking)  Val2240  Met2345 | Leu2185  Tyr2225 (cation-π stacking) |
| CHEMBL1180264 | Leu2185  Lys2187  Ile2237  Ile2356  Asp2357 | Ile2167  Pro2169  Glu2190  Leu2192 |
| CHEMBL165714 | Leu2185  Ile2237  Trp2239  Val2240  Ile2356 | Lys2187  Tyr2225  Cys2243  Met2345 |
| CHEMBL1621113 | Asp2195  Tyr2225 (cation-π stacking)  Ile2237  Asp2357 | Gln2167  Pro2169  Leu2185  Lys2187  Ile2356  Phe2358 |
| CHEMBL1079062 | Leu2185  Trp2239 (π-π stacking/cation-π stacking)  Val2240 | Met2345  Ile2356 |
| CHEMBL267516 | Leu218  Gln2194  Asp2195  Trp2239 (cation-π stacking)  Ile2356 | Leu2192  Ile2237  Met2345  Asp2357 |
| CHEMBL1545778 | Lys2187  Asp2195  Ile2237  Tyr2225 (π-πstacking)  Val2240  Ile2356  Asp2357 | Glu2190  Leu2192  Met2345 |
| CHEMBL1366164 | Leu2185  Trp2239 (cation-π stacking)  Val2240  Met2345  Ile2356 | Ile2163  Pro2169  Lys2185  Cys2243 |
| CHEMBL1642794 | Ile2163  Leu2185  Tyr2225 (cation-π stacking)  Ile2237  Trp2239 (π-π stacking)  Val2240  Thr2245  Ile2356 | Pro2169  Met2345  Ser2342 |
| CHEMBL2165395 | Tyr2225 (cation-π stacking)  Trp2239 (cation-π/π-π stacking)  Val2240  Met2345 | Ile2163  Leu2185  Gly2238 |
| CHEMBL45196 | Lys2187  Glu2190  Leu2192  Asp2195  Asp2357  Phe2358 (π-π stacking) | Gln2194  Ile2237  Gly2359 |
| CHEMBL2143987 | Trp2239 (π-πstacking/cation-π stacking)  Ser2342 | Ile2163  Pro2169  Leu2185  Thr2245  Met2345 |
