# Supplementary material for: Mimetics of extra virgin olive oil phenols with anti-cancer stem cell activity
Source: Aging (Albany NY). 2020 Nov 9;12(21):21057–75. doi: 10.18632/aging.202154 (PMC7695371; doi:10.18632/aging.202154)
Supplement: Supplementary Table 7 [file aging-12-202154-s004..docx]

**Supplementary Table 7.** **Key interacting residues of oleacein mimetics to the catalytic site of DNMT.** Interactions other than electrostatic are highlighted in yellow (possible) or green (reliable).

| Oleacein mimetic | Main catalytic residues | Other relevant residues |
| --- | --- | --- |
| CHEMBL2172394 | Asn1578  Pro1225  Asn1267 | Gly1223  Ala1579  Ser1146  Glu1266  Val1268  Arg1310  Phe1145 (π-π stacking) |
| CHEMBL1085246 | Asn1578  Ala1579  Val1580  Phe1145  Ser1146  Gly1147  Gly1223  Pro1224  Pro1225  Asn1267 | Cys1148  Glu1266 |
| CHEMBL357073 | Val1580  Phe1145 (π-π stacking)  Ser1146 | Asn1578  Ala1579  Leu1151  Pro1225 |
| CHEMBL1632504 | Ala1579  Val1580  Phe1145  Ser1146  Gly1147  Leu1151  Glu1168 | Asn1578  Trp1170  Gly1223  Met1169  Pro1225  Glu1266 |
| CHEMBL126593 | Ala1579  Val1580  Phe114  Ser1146  Gly1150  Pro1225 | Asn1578  Gly1147  Cys1148  Leu1151  Glu1168  Gly1219  Pro1224  Leu1247 |
| CHEMBL1950046 | Ala1579  Val1580  Asp1143  Ser1146  Gly1150  Leu1151 | Asn1578  Gly1147  Glu1266  Ser1152 |
| CHEMBL1440472 | Asn1578  Ala1579  Gly1147  Glu1168  Gly1223  Pro1225  Trp1170 (halogen bond) | Phe1145  Ser1146  Gln1575  Val1580  Cys1148  Gly1150  Leu1151  Pro1224 |
| CHEMBL1300434 | Val1580  Phe1145  Ser1146  Glu1168  Trp1170  Pro1225 | Asn1578  Met1169  Gly1226  Glu1266  Ala1579  Gly1147  Asn1267 |
| CHEMBL1890048 | Phe1145  Gly1147  Glu1168  Trp1170 (π-π stacking) | Gln1575  Arg1574  Ala1579  Ser1146  Met1169  Pro1225  Leu1247 |
| CHEMBL1180264 | Asn1578  Phe1145  Glu1266  Ala1579  Ser1146  Gly1147 | Met1169  Arg1574  Val1580  Leu1151  Ser1146  Trp1170  Gly1173 |
| CHEMBL165714 | Val1580 | Ala1579  Pro1225  Asn1267  Asn1578  Arg1147  Ser1146  Gly1150  Trp1170  Gly1223  Pro1224  Glu1266 |
| CHEMBL1621113 | Asn1578  Gly1223  Asn1267  Pro1225 (π-π stacking)  Pro1224 (halogen bond) | Glu1266  Arg1310  Arg1312  Ala1579  Val1580  Phe1145  Ser1146  Gln1227  Val1268 |
| CHEMBL1079062 | Asn1578  Ala1579  Val1580  Gly1150  Leu1151  Gly1223  Glu1266  Asn1267 | Ser1146  Gly1146  Pro1225  Arg1310 |
| CHEMBL267516 | Ala1579  Met1169  Gly1223  Pro1225  Trp1170 (π-π stacking) | Glu1168  Asn1578  Phe1145  Ser1146  Gly1147  Pro1224  Leu1247  Glu1266  Asn1267 |
| CHEMBL1545778 | Asn1578  Phe1145 (π-π stacking)  Ser1146  Gly1222  Gly1223  Pro1225 | Val1580  Pro1224  Ala1579  Val1144  Leu1151  Met1169  Leu1247 |
| CHEMBL1366164 | Asn1578  Ser1146  Gly1223  Pro1225  Glu1266  Phe1145  Pro1224 (π-π stacking) | Val1580  Asn1267  Ala1579  Leu1151 |
| CHEMBL1642794 | Asn1578  Phe1145  Pro1225 | Ser1146  Ala1579  Val1153  Gly1223 |
| CHEMBL2165395 | Ala1579  Val1580  Phe1145  Ser1146  Gly1147  Leu1151  Glu1168  Met1169  Trp1170 (halogen bond) | Gly1150  Gly1223  Pro1225  Arg1147  Asn1578  Leu1247 |
| CHEMBL45196 | Gln1575  Asn1578  Gly1147  Glu1168  Trp1170 | Arg1574  Ala1579  Met1169  Phe1148  Ser1149  Cys1148  Gly1150  Ala1173  Gly1223  Pro1225  Leu1247 |
| CHEMBL2143987 | Asn1578  Ala1579  Val1580  Gly1150  Gly1223  Pro1225  Asn1267 | Ser1146  Phe1145  Gly1147  Cys1148  Leu1151  Pro1224  Glu1266  Val1268 |
